# Wetting Properties of Simulated and Commercial Contaminants on High Transmittance Superhydrophobic Coating

**DOI:** 10.3390/nano13182541

**Published:** 2023-09-11

**Authors:** Michele Ferrari, Francesca Cirisano

**Affiliations:** CNR-ICMATE National Research Council, Institute of Condensed Matter Chemistry and Technologies for Energy, Via De Marini 6, 16149 Genova, Italy

**Keywords:** wettability, superhydrophobic surface, solar panels, agrosolar, bird droppings, transmittance, energy

## Abstract

The large and necessary diffusion of huge solar plants in extra urban areas implies the adoption of maintenance strategies especially where human intervention would require high costs and logistic problems. Animal dejections like bird droppings and agricultural sprays are environmental agents able to significantly decrease light absorption and, in some cases, cause serious damage to the electric conversion systems in a photovoltaic panel. In this work, the performance of a superhydrophobic (SH) coating in terms of durable self-cleaning properties and transparency has been studied in the presence of commercial and simulated contaminants on glass reference and solar panel surfaces. Wettability studies have been carried out both in static and dynamic conditions in order to compare the compositional effect of commercial liquids used as fertilizers or pesticides and molecules like pancreatin as model substances simulating bird droppings. From these studies, it can be observed that the superhydrophobic coating, independently from the surface where it is applied, is able to repel water and substances used such as fertilizers or pesticides and substances simulating bird droppings, maintaining its properties and transparency. This kind of approach can provide information to design suitable spray formulations without the above-mentioned drawbacks to be used in natural environment areas and agrosolar plants.

## 1. Introduction

Clean and transparent coatings are strongly required in many technologies where efficient light capture and/or constant optical properties are essential but often endangered by external agents [1]. The search for larger, suitable locations for solar plants outside urban areas raises new issues related to environmental pollutants potentially affecting the physicochemical properties of such surfaces. The worldwide growth and very recent diffusion of photovoltaic plants in agriculture (agrosolar) [2,3] represent a new deal toward a green society hopefully more independent from fossil fuels. In such environmental situations, solar panels then undergo unknown conditions and their efficiency will need to be tested. In fact, the presence of dispersant agents like surfactants in fertilizers or pesticides and animal life can produce, by impacting on a surface, significant contamination by adsorption or adhesion, critically influencing the shelf life of a solar panel surface and compromising its durability and functionality. The growth of agrosolar has fostered the investigation of the effects of commercially selected few categories of products used in agriculture as a spray so that their effect in conditioning the surface of solar panels cannot be neglected. Solar panels are covered by a hydrophobic layer whose degree of water and organic liquid repellence does not allow a self-cleaning action and requires human intervention to restore, even partially, the initial performance. In such conditions, both in industrial or rural areas, dust, powders or pollens can accumulate daily with a dramatic loss in the yield of the plant [4].

New solutions consisting of coatings with superhydrophobic/amphiphobic properties have been successfully applied to provide self-maintaining systems for reducing or avoiding human intervention, especially in large plants or harsh environments to keep the original functionalities [5,6]. Amphiphobicity is a feature of a surface showing both the oil and water repellence. In particular, such surfaces result from a combination of low surface energy material with a specific surface morphology (micro/nanoroughness) [7]. From the wettability point of view, a surface can be defined as hydro/oleophilic when water or oil contact angle (CA) is <90°, hydro/oleo phobic when CA > 90° and superhydro/oleo phobic when CA > 150°.

In numerous technological and industrial applications, such as anti-icing [8], ink-jet printing [9], spray cooling [10] and pesticide and herbicide delivery [11], the phenomena correlated to liquid droplet impact with a solid surface play a key role. When a droplet impacts a solid surface, it can deform by wetting the surface, retracting or bouncing. The type of phenomenon that will occur depends on how much kinetic energy the droplet loses during the impact as it is transformed into surface energy as a consequence of the increase in surface area. This is then associated with the type of surface material, and therefore, its surface energy, one of the most influential parameters, with which the liquid will come into contact. On surfaces with low surface energy, such as superhydrophobic surfaces (SHS), the impact will produce a minimum dissipation of energy causing the droplet to rebound. Generally, the lower the kinetic energy of the drop, the better the phenomena of restitution and rebound; as the kinetic energy increases, droplet fragmentation during bouncing occurs. The drop impact dynamics on SHS and the study of the emerging phenomena is the subject of numerous recent studies. In a general perspective, the authors consider various parameters to explain their observation such as contact time, liquid viscosity, dissipation energy, surface elasticity, impact velocity, number of rebounds and coefficient of restitution and usually they are correlated and evaluated throughout the Weber number (*We*), a parameter depending on the impact velocity and liquid properties. As an example, Nakajima et al. in [12] have studied how the nature of the hydrophobic substrate and its rigidity influence the sliding behavior of water droplets. They found that a *v* = 0 (*We* = 0), the more rigid surface presents a low sliding velocity (higher the energy barrier due to the rigidity), increasing speed and We a similar sliding velocity value is reached until for *We* = 43 when the order reversed, the rigid surface shows higher sliding velocity with respect to the flexible surface. In conclusion, they found that the order of dynamic hydrophobicity depends on the presence of certain initial impact velocities and on the solid surface’s physical and chemical characteristics. A similar study but on an elastic nanostructured superhydrophobic surface was conducted in [13] where the authors investigated the impact dynamics of water droplets on substrates having different stiffness. In this work, contact time, impact velocity and We were considered. We found that, for the same impact velocity, a droplet on a rigid surface shows breakup and splashing (high contact time) compared to an elastic substrate where the drop is deformed in a pancake-like shape reducing its contact time due to the oscillation of the substrate imparting a vertical momentum allowing bouncing.

Hao et al. [14] and Quan et al. [15] have studied the dynamics of water droplets impinging on the surface with different microstructure and roughness. In both studies, it was found that the impact velocity is important to define the wetting transition of the SHS (Cassie–Baxter to Wenzel state) but the transition is also strongly influenced by the surface micro-nano structure that is responsible for the amount of air trapped between micropillar. A substrate with a hierarchical structure is able to sustain higher dynamic liquid pressure (high impact velocity) reducing liquid contact time and promoting rebound. As reported, velocity is often used to describe the surface behavior and in parallel, as mentioned above, the Weber number can describe the type of drop impact and deformation. In particular, it was found that, for low velocity (0.2–2 m/s) and We, drops bounce with low deformation during the impact and the contact time is independent of impact velocity. The contact time, on the other hand, is dependent on the drop dimension and the drop deformation after impact increases linearly with velocity [16,17]. Furthermore, Richard et al. [18] also observed that a low impact velocity does not affect the coefficient of restitution for pure water and water/glycerol mixture. To study the influence of liquid properties on spreading and bouncing phenomena, water-based model systems have been used by some authors. Deng et al. [19], for example, have used ethanol-water and glycerol-water drops to investigate the role of viscosity and surface tension in wetting phenomena on superamphiphobic surfaces. They found, as seen from the previously cited works, that at a low impact velocity (0.35 m/s), the contact time was constant and independent from liquid properties but by increasing the velocity up to 2 m/s the contact time increased. This fact is probably due to the superamphiphobic nature of the surface which is different from the only superhydrophobic situation. Another observation at low velocity is that the restitution coefficient decreases with the increase in liquid viscosity and increases with the surface tension. Jha et al. [20] arrived at the same observations using solutions of water and glycerol with different viscosities but constant surface tension. As reported before, researchers use water or at most water mixtures (ethanol, glycerol), to vary the properties of the liquid as liquids for their studies on SH surfaces. This obviously simplifies the already complex system, but at the same time provides partial models and results. High water and oil-repellent properties are then required in those fields like renewable energy to increase the yield and decrease the modules’ degradation. Among these solutions, amphiphobic coatings can represent a way to prevent pollution from dust accumulation, water and oil-based natural and urban aerosols, from agriculture dispersions to bird droppings [21]. Still, many open issues have to be addressed and key surface aspects have to be faced for keeping the optical features of transparent surfaces. Even if it is not extensively investigated, the automotive field provides few references dedicated to the effect of bird droppings on clear coats during a car service life. Among several biological fluids causing possible local defects in the paint of the car body, in this study [22] the authors aim to reveal the mechanism of degradation of bird droppings by comparing clear coats at different compositions. They created the conditions for a local etching of the surface via a hydrolysis reaction of coating polymer by using pancreatin as a synthetic model substitute of natural products to simulate the effect of a digestive enzyme (lipase) present in bird droppings. In a similar study [23], the authors investigated the catalyzed hydrolytic degradation on clear coats under the effect of natural and simulated (pancreatin) bird droppings using different analytical techniques. The presence of highly etched areas on the surface was interpreted by different mechanisms including acid, metal ion catalysis and enzymatic catalysis finding a weak contribution to catalyze the hydrolysis reaction of clearcoat by acid and metal ions, while enzymes existing in bird droppings were found responsible for the degradation. Again, the effect of clearcoat composition has been studied in [24] reporting the preparation of acrylic/melamine-based formulation containing up to 8 wt% of a reactive polysiloxane additive. The resistance against bird droppings was enhanced, and its surface properties were studied by contact angle measurements, ATR spectroscopy and energy-dispersive X-ray analysis. The coating properties underwent exposure to pancreatin, the synthetic equivalent of natural bird droppings. Beyond the improved effectiveness of the additive reducing the coating surface free energy, the work underlined some limitations at higher concentrations with phase separation leading to an undercure problem in its finishing state. The same topic has been investigated in [25] studying the effect of bird dropping in marine environments focusing on the damage produced by their contaminant content on the boat textile, finishing composition and surrounding water. The interaction between bird droppings and rain is discussed in [26] where the authors studied their influence on the determination of rain chemical composition in precipitation methods. A biomimetic approach has been followed in order to find an efficient way to reduce adhesion to limit insect crawling. Organic-inorganic coatings with multi-scaled roughness and particle-transferring surfaces have been compared with natural examples of non-adhesive and slippery systems [27]. The accumulation of natural and artificially prepared dust of different sizes and morphologies was investigated in [28] for studying the power loss in solar modules. A linear dependence with the deposition density coming from atmospheric aerosols was found in relation to the tilt angle of the module, the period of exposure, conditions of local climate, wind movement and dust properties. Similarly, in [29] dry and rainy periods have been compared in the loss in power efficiency due to optical performance decrease due to soiling and dust accumulation. In this study, an automatic cleaning system was efficiently employed in the overall annual energy recovery. A high potential source of surface contamination is offered by the worldwide use of surfactants in agriculture aimed to improve efficiency in the leaves uptake for those substances like pesticides or herbicides for protective, growth and defoliant action. Their use as adjuvants is necessary to tailor specific properties in wetting actions or to stabilize emulsions and dispersions in preparation for spray application [30]. The enhanced wetting potential of such products can result in a permanent contamination layer on solar panels, in combination with other substances like inorganic powders, pollens and so on. The growing interest in eco-friendly formulations has pushed some authors to investigate essential oils in oil-in-water (o/w) emulsions stabilized by an amphiphilic copolymer in relationship to their potential insecticidal activity [31]. Moreover, the geographical position can influence the investigations on local environments with a wide range of surface-active substance by-products from human presence from urban to rural sites [32,33] and local cultural habits without strict environmental laws usually hold to unpredictable groups of pollutants like a fingerprint of a specific area. The site-specific atmospheric chemistry can then require tailored cleaning requirements in view of maintaining the power yield [34,35]. For example, the presence of anionic surfactants in atmospheric aerosols can be derived from soot degradation coming from diesel fuels. In this study [36], colorimetric techniques have been applied to detect anionic surfactants as the main component concentration of finer aerosols, while aqueous extracts of such aerosols significantly reduced the surface tension.

For this reason, in this work, the authors studied the effects of simulated and anthropogenic pollutants on the surface and optical properties of highly hydro and oleophobic coating. In previous works, the durability of such coating has been investigated in on-field tests in real conditions of urban polluted areas [37]. The results show the effectiveness of the combination of roughness and composition in the protection of a surface in solar panels from water-based formulations and providing information for the preparation of agricultural sprays with a lower impact on solar plant maintenance procedures.

## 2. Materials and Methods

The mixed organic–inorganic superhydrophobic/amphiphobic coating was prepared on laboratory soda lime glass and on a commercial solar panel surface by spray coating technique applying the dispersion of fumed silica nanoparticles (EVONIK HDK H15 with agglomerate 1–250 um, primary particles 5–30 nm) at a concentration of 2 g/L in a fluoropolymer blend solution of a fluorosilane polymer (0.1 wt.%) carried in a hydrofluoroether solvent, methoxy-nonafluorobutane [37]. The prepared coating shows a silica quantity of 0.008 mg/cm^2^. To study the surface behavior against different substances, three liquids used in agricultural applications and a simulated bird droppings solution, in addition to the water, were employed. In particular, they are:

(A) fertilizer NPK 6-5-5, water-based dispersion of salt of nitrogen (present as nitric, ammonia and ureic salts), phosphorous and potassium (N tot 6%, P_2_O_5_ 5%, K_2_O 5%);
(B) linseed oil 0.2%, for the biological/organic treatment of plants against insects, especially the cochineal;
(C) broad spectrum liquid insecticide in aqueous emulsion (2.5 g/L) based on technical permethrin (2.5 g/L);
(D) pancreatin water dispersion (100 g/L) used as a model substance in substitution for bird droppings [24].

No mixtures between the previous liquids have been investigated in order to focus and better observe the behavior of every single preparation, often a mixture itself, and then to avoid any mutual influence.

The surface tension (γ) and contact angle measurements were performed by the ASTRAview tensiometer (developed at CNR–ICMATE [38]) in a thermostatic chamber at 20 °C and under saturated vapor conditions in order to avoid a volume decrease due to the evaporation process. In addition to careful monitoring of any drop volume change, ASTRAview allows the drop size to be followed at each measurement stage. A stainless-steel capillary with a diameter of 0.70 mm connected to a syringe was used to produce an 8 µL drop in order to be deposited on the solid surface for the CA measure or to obtain the surface tension value. The data were collected out of 3 to 5 sets of acquisition in different positions of the surface showing reproducibility of 1°, thus proving the homogeneity of the coating. Contact angle hysteresis (CAH) was measured as the difference between the advancing and receding contact angles using frames captured with the high-speed camera. The evaluation was conducted on droplets falling on a surface tilted by 3°, this value was chosen as it is ten times smaller than the value at which solar panels are normally installed at 30° [39]. The obtaining of data regarding the number of bounces of five different liquids was performed using a high-speed camera at 3500 fps (Sprinter HD, Optronis, Kehl, Germany) deposited on a flat surface. To produce water droplets of about 5 µL, a stainless-steel capillary with a diameter of 0.21 mm was connected to a syringe from a height of 20 mm (tip to surface). The surface morphology of the SHS was observed and evaluated by 3D confocal and interferometric profilometric measurements (Sensofar S-NEOX, Barcelona, Spain) and surface roughness (Sa), an amplitude parameter was acquired according to the standard ISO 25178 [40]. Finally, the average roughness was reported as the sum of absolute values of data differences from the mean. 

The transparency of the coating, by transmittance measurements (UV-VIS spectrometer, Ocean Optics Flame Spectrometer, Halma Company, Amersham, UK), was measured as reported in [37] and by the same procedure, the transmittance of the selected liquids was evaluated to understand their absorption properties and evaluate their impact on the overall panel yield.

Furthermore, in this study, the Weber number (*We*) was used to understand the wetting behavior of the surface with respect to the different used liquids. Weber number is a dimensionless quantity describing the ratio of fluid inertia to surface tension given by $We=\rho v^{2}D_{0}/\gamma ,$ with *ρ* the density, *v* the velocity, *D*_0_ the diameter of the droplet, *γ* the surface tension. Despite the Weber number representing the competition and the balance between the surface tension and the kinetic energy of spreading in our case, being velocities and densities quite similar in a narrow range, the surface tension effect is the prevalent parameter to be taken into consideration.

Finally, *We* was correlated with other parameters such as spreading factor (*β*), restitution coefficient and contact time.

## 3. Results and Discussion

### 3.1. Surface Characterization

**Figure 1:**
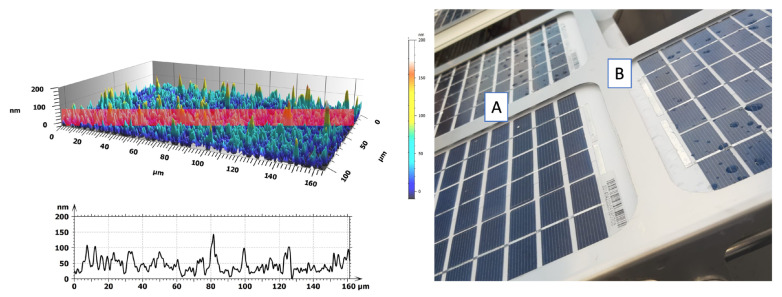
On the left, 3D image of sample and correlated roughness profile acquired by 3D interferometric and confocal profilometer of SH coating with Sa = 52 ± 4 nm, on the right a photo of coated (**A**) and uncoated (**B**) solar panel for field experiments.

The prepared superhydrophobic coating was characterized by wettability and surface morphology measurements to assess its homogeneity and properties. In particular, by water CA measurements it was observed that the surface is strongly SH, with drops showing CA > 170°, with the lowest surface energy (<15 mN/m) and with ultra-low adhesion as a result of the bouncing study. By 3D confocal and interferometric profilometric measurements, it was observed that the surface is extremely homogeneous with an average roughness of 52 ± 4 nm (Figure 1). Furthermore, its transmittance was estimated by measuring the light transmission in the range UV-Vis between 350 nm and 750 nm in transmission mode by UV-VIS spectrometer at room temperature and the prepared SHS shows a transmittance of about 94% [37].

### 3.2. Wettability and Liquids Behaviours

All liquids used in this experiment (A–D and water) were characterized by measuring their surface tension (γ), density (ρ) and for each one the wettability, contact angle on SH surface (CA_SHS_) and on the untreated solar panel surface (CA) and contact angle hysteresis (CAH) on the SH surface have been estimated. CAH on an untreated surface results in higher than 15° for each liquid. The data are reported in Table 1.

**Figure 2:**
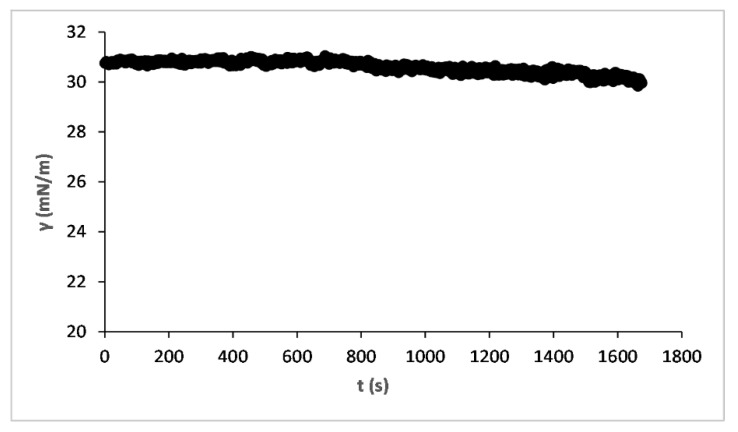
Surface tension dynamics of liquid C.

As from Table 1, the components of liquids do not have strong surface active action except for liquid C where the presence of surfactants, to ensure an optimal and efficient wetting effect on plants, even unknown, can be present in concentration probably over the critical micellar concentration (cmc) [41]. This assumption, concentration over the cmc, is supported by surface tension measurement of liquid C for a long time: it is possible to observe in Figure 2 that the value is steady in the studied time window since the early adsorption stage. If the liquid had not already reached the cmc, we would observe surface tension dynamics in the first moments of measurement, an unobserved trend in our measurements that confirms our assumption.

**Figure 3:**
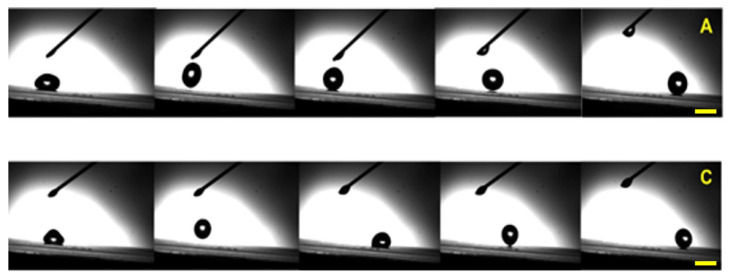
Slow motion sequence acquired by the high-speed camera at 3500 fps (Sprinter HD, Optronis) of liquid A and C droplets impacting with a tilted (3°) SH coating. Scale bar 2.5 mm.

Impact studies by high-speed cameras have been carried out to assess more dynamic properties of the liquids in contact with SHS. All liquids do not have an appreciable wetting action (CA > 150°) on the prepared coating, in fact, contact angle hysteresis is very low in all the cases except for liquid C showing a more enhanced wetting action in dynamic conditions (higher CAH) during the first impact. The behavior of liquid C can be explained by considering the adsorption phenomena at the liquid-solid interface due to surfactants in it. Nevertheless, all substances assess the effectiveness of the SH coating in repelling water-based or oil-in-water formulations in fact a very low tilt angle (<3°) is enough to avoid the drop adhesion at very short times as reported in Figure 3 for liquid A and C. The use of a tilt angle at a higher inclination, as in real solar panel installation, the effect is even more pronounced making the coating’s high performance evident.

**Figure 4:**
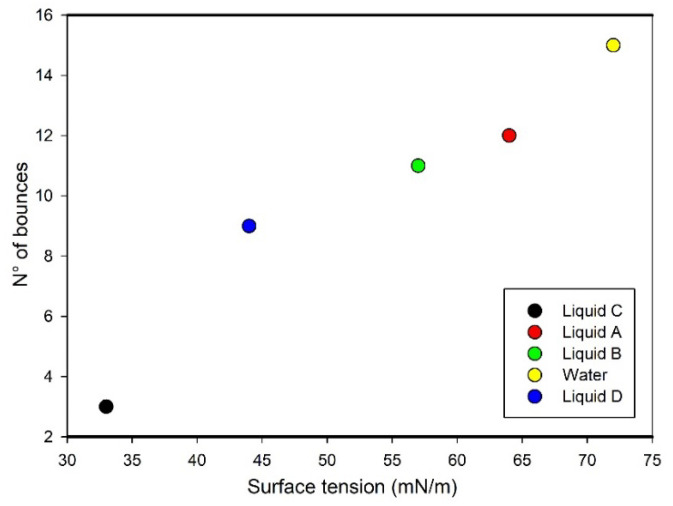
Number of bounces of the five different liquids on the superhydrophobic surface from height of 20 mm (tip to the surface).

The way liquids interact with the SHS was observed and discussed analyzing high-speed videos able to capture the number of bounces for each liquid. In Figure 4 the number of bounces vs. the surface tension is reported.

The number of bounces linearly decreases with the surface tension keeping an enhanced SH effect up to intermediate values while, in the presence of surface-active components, the number dramatically lowers.

As expected from the surface tension value, water shows the highest number of bounces while liquid C, with the lowest surface tension and high CAH, due to its surface activity, results in a significantly lower number of impacts.

In the case of liquid D, pancreatin (enzyme with protein structure), the adsorption effect at the liquid-solid interface with SHS seems to be neglectable in the experimental time window (high CA and low CAH) because of the short impact time. On the other side on an untreated surface, the higher adhesion properties could allow in addition a drop spreading effect with covering a larger area.

**Figure 5:**
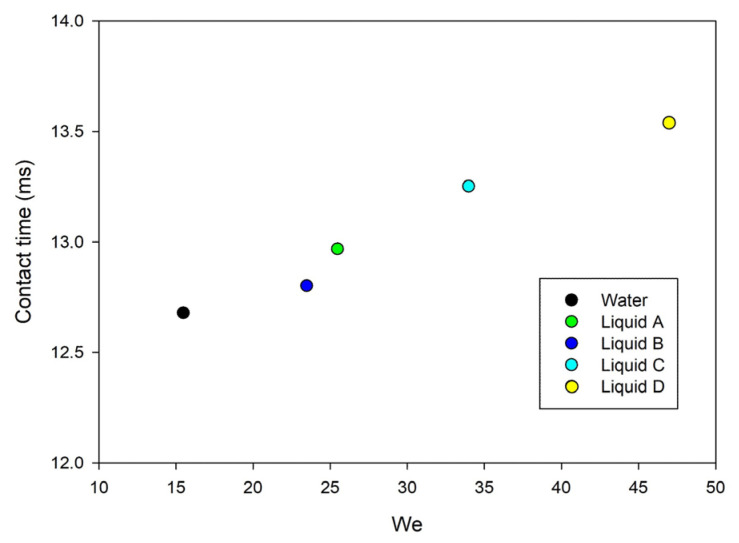
Contact time vs. We of the tested liquids.

Drop contact time can be an additional parameter for following the evolution of drop dynamics and its dependence on *We* is reported in Figure 5. Considering that, all the liquids have similar densities and velocities, *We* underlines the role of surface tension in determining a linear behavior. Moreover, all the liquids result in a contact time range of 1 ms, indicating that even with a greater span of surface tension the superhydrophobic behavior is maintained.

**Figure 6:**
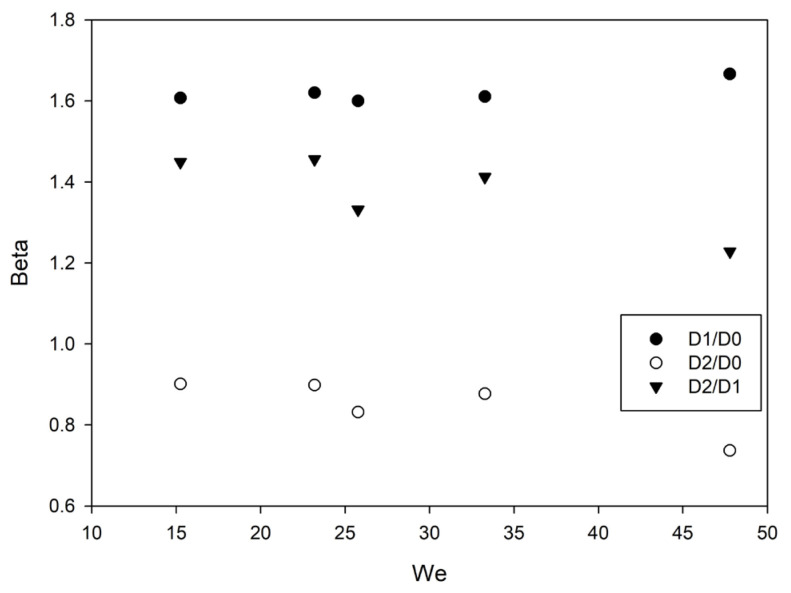
Spreading factor (*β*) vs. Weber number (*We*) of the used liquids in the first two impacts (*D*_1_/*D*_0_, *D*_2_/*D*_0_) and between the two impacts (*D*_2_/*D*_1_).

Despite their smoothness in terms of nano-micro roughness and control of the inorganic contribution only to a certain extent, on such heterogeneous surfaces, after the impact, the liquid drop can undergo pinning or impinging phenomena potentially affecting both the advancing and the receding motion. For this reason, the diameters after the first two impacts have been measured and the consequent spreading factor compared to the *We* number (Figure 6).

The spreading factor (*β*) is defined as the ratio between the maximum diameter (*D*_1_, *D*_2_) of the liquid after the first and second impact with the surface to the initial droplet diameter (*D*_0_) before its contact with the solid material. This parameter allows a description of drop deformation during the impacts according to liquid-solid interactions and liquid properties.

Under the most dynamic conditions as in the first two impacts, the drop diameter variation (*D*_1_/*D*_0_, *D*_2_/*D*_0_) appears to be constant in a quite wide *We* range despite a significant, but expectable, decrease in contact time. Impinging phenomena are seemingly not present in these experiments showing a linear retracting motion after the impact. As expected at this low-velocity range, drops rebound and retract without pinning or formation of smaller satellites [14,19].

The slight slope after the second rebound (*D*_2_/*D*_1_) indicates that, despite the energy loss, even at a very small impact velocity, the oleophobicity is maintained.

**Figure 7:**
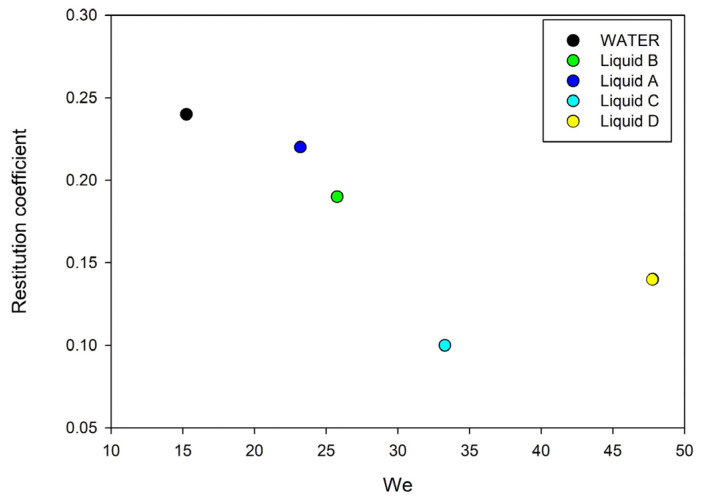
Restitution coefficient vs. Weber number of the used liquids.

Contact angle analysis can provide further insight into the retraction regimes for the restitution coefficient. This parameter is defined as the ratio between the velocity after and before an impact and can represent a picture of the effect of potential drop impalement caused by viscous dissipation inside the coating roughness. Considering the liquid velocities as comparable, the Weber number strongly depends on surface tension and density as in Figure 7.

At this stage, it is difficult to define the degree of impalement in such heterogeneous surfaces. Samples show spreading dynamics not influenced by impalement according to the advancing angle while in the retraction stage, the presence of surface active and adsorption phenomena is reflected by a more evident receding angle. In the cases under investigation, drop-surface interaction parameters seem to be driven by the liquid surface properties more than the bulk ones. Even where they have been used as in the *We* number the surface tension always plays a significant role.

The complementary part of this study is related to the effect of the liquids under investigation on the optical properties of transparent coatings. The potential light absorption of liquid drops deposited on the surface can significantly affect the yield of the solar panel efficiency [21]. For this reason, the transmittance of every liquid sample has been measured; a not negligible, if not dramatic, decrease in the light transmission was found supporting the use of SH coating to protect solar panels.

**Figure 8:**
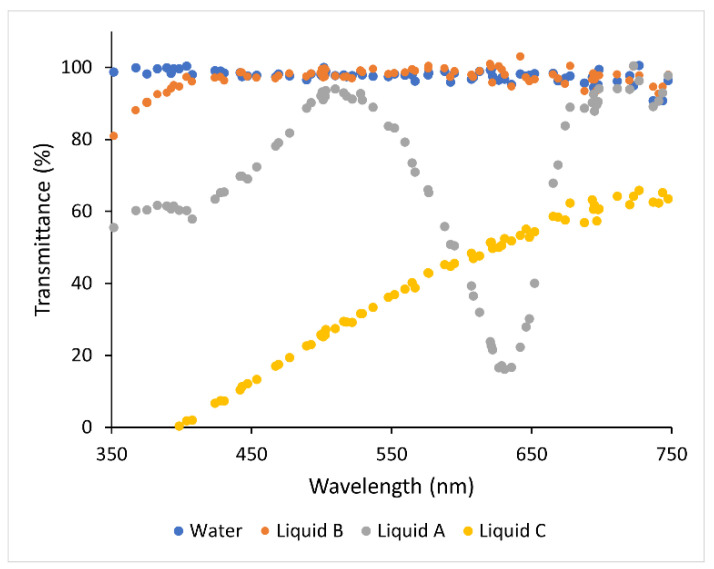
Optical transmittance as a function of wavelength (nm) for the different tested liquids.

In Figure 8 it is possible to observe:

- in liquid A the presence of coloured salts significantly alters the transmittance of the liquid film according to a specific wavelength range.
- in liquid B the linseed oil dispersion does not affect the transmittance with a quantitative signal comparable with the water one in the range of wavelength studied.
- in liquid C the presence of surfactant (unknown composition as a commercial product) results in significant absorption mainly in the near UV region due to the potential presence of aromatic rings or unsaturated chains and an overall decrease in transmittance is observed.
- for liquid D, the signal is not reported because, even with surfactant samples at a lower concentration, pancreatin dispersion completely hinders the suitable light transmission.

These data suggest that a special wettability of the solar panel surface is important to prevent the deposition of substance, also water-based, that remaining and drying on the surface can reduce the transmission of solar energy, reducing the efficiency of the panel itself.

## 4. Conclusions

In this work, we have investigated the behavior of SH coatings in view of their application in areas where pollution could be derived from the extensive use of agricultural spray like in recently growing agrosolar plants. At this site the solar panel surfaces are exposed to substances other than those derived from urban areas but, at the same time, they potentially can significantly and irreversibly modify the surface properties preparing them for a more consistent accumulation of environmental contaminants. From this study, SH coating can be applied to different substrates (laboratory glass and solar panel surface) with high repellent properties against liquids of different nature like insecticides, fertilizers and pancreatin as a bird-dropping model. In particular, in this work, we investigated which negative potential such substances could have as contaminants, conditioning the surface for further and more persistent degradation effect of its properties. Physicochemical characterization and utilization parameters like contact time, *We* number, surface tension and finally the transmittance directly influence the photon absorbance. In conclusion, we can affirm that the use of a superhydrophobic coating can limit, if not avoid, the inconvenience brought by the liquids used in agriculture significantly affecting the solar panel yield because of their adhesion and adsorption on the panel surface.

These results obtained can address the formulation design of agricultural sprays toward a lower, if not absent, content of surfactants both for their significant potential light absorption and for their wetting enhancing effect on the panel conditioning the surface allowing dust or other pollution agent to be set on the panel requiring, when possible, an important maintenance action. The behavior of linseed oil dispersion, liquid B, has been proved to provide optimal performance in terms of minimum light absorption (almost absent) and in terms of very low wetting properties on SH coating. In conclusion, the presence of an SH coating with a self-cleaning effect represents a complementary component in a panel considering the worldwide diffusion of huge solar plants especially in desert areas or outside urban sites where maintenance could require daily, man-powered intervention.

## Figures and Tables

**Table 1:** Surface tension (γ), density (ρ), contact angle on SHS (CA_SHS_), contact angle on untreated solar panel (CA) and contact angle hysteresis on SHS (CAH) of the five used liquids.

Liquid	γ (mN/m)	ρ (g/cm^3^)	CA (°)	CA_SHS_ (°)	CAH (°)
Water	72	1.0	51	>170	1
A	64	1.45	62	164	1
B	57	1.28	38	159	1
C	31	1.20	33	157	65
D	44	1.39	41	158	1

## Data Availability

The data presented in this study are available on request from the corresponding authors.
